# Parvalbumin-, substance P- and calcitonin gene-related peptide-immunopositive axons in the human dental pulp differ in their distribution of varicosities

**DOI:** 10.1038/s41598-020-67804-x

**Published:** 2020-06-30

**Authors:** Sook Kyung Park, Seung Ki Choi, Youn Gyung Kim, So Young Choi, Jin Wook Kim, Sang Hyeok Seo, Ji Hyun Lee, Yong Chul Bae

**Affiliations:** 1 Department of Anatomy and Neurobiology, School of Dentistry, Kyungpook National University, 188-1, 2-Ga, Samdeok-Dong, Jung-Gu, Daegu, 41940 Korea; 2 Department of Oral and Maxillofacial Surgery, School of Dentistry, Kyungpook National University, Daegu, 41940 Korea

**Keywords:** Dental pulp, Somatic system

## Abstract

Information on the frequency and spatial distribution of axonal varicosities associated with release of neurotransmitters in the dental pulp is important to help elucidate the peripheral mechanisms of dental pain, mediated by myelinated versus unmyelinated fibers. For this, we investigated the distribution of axonal varicosities in the human dental pulp using light- and electron-microscopic immunohistochemistry for the vesicular glutamate transporter 2 (VGLUT2), which is involved in the glutamatergic transmission, and syntaxin-1 and synaptosomal nerve-associated protein 25 (SNAP-25), combined with parvalbumin (PV), which is expressed mostly in myelinated axons, and substance P (SP) and calcitonin gene-related peptide (CGRP), which are expressed mostly in unmyelinated axons. We found that the varicosities of the SP- and CGRP-immunopositive (+) axons were uniformly distributed throughout the dental pulp, whereas those of PV+ axons were only dense in the peripheral pulp, and that the expression of PV, VGLUT2, syntaxin-1, SNAP-25, SP and CGRP was significantly higher in the varicosities than in the axonal segments between them. These findings are consistent with the release of glutamate and neuropeptides by axonal varicosities of SP+ and CGRP+ unmyelinated fibers, involved in pulpal pain throughout the human dental pulp, and by varicosities of PV+ fibers, arising from parent myelinated fibers, and involved in dentin sensitivity primarily in the peripheral pulp.

## Introduction

The dental pulp is densely innervated by nociceptive neurons; the release of glutamate by their axons is a crucial part of the mechanism of signaling of acute nociceptive pain and of pathological pain^1–4^. The large majority of sensory neurons in the trigeminal ganglion (TG) that innervate the dental pulp give rise to myelinated (A) fibers and a few give rise to unmyelinated (C) fibers^5^. While the myelinated fibers are involved in the signaling of sharp, pricking, well-localized pain originating from the dentin and the peripheral pulp, the unmyelinated fibers are involved in signaling of dull, achy, poorly-localized pain originating from the pulp itself; the former play an important role in the dentin sensitivity, the latter play an important role in pulpal inflammatory pain^6,7^.

The pulpal axons frequently exhibit multiple varicosities along their length^8–11^, short segments where the axon is enlarged and contains multiple vesicles and mitochondria^12,13^. They also express two classes of proteins, which are involved in the loading of glutamate into synaptic vesicles and its release, the vesicular glutamate transporters (VGLUT) and the soluble *N*-ethylmaleimide sensitive factor attachment protein receptors (SNARE)^9,10,14–16^. These findings suggest that pulpal axons release glutamate from their varicosities. If this hypothesis is correct, it becomes important to collect information on the frequency and spatial distribution of varicosities of pulpal axons arising from parent myelinated axons and unmyelinated axons, and correlate that to what is known about their role in mediating nociception.

For this, we here investigated the distribution of varicosities in the human dental pulp from human premolar teeth using light microscopic immunohistochemistry for VGLUT, combined with parvalbumin (PV), which is expressed mostly in parent myelinated axons, and substance P (SP) and calcitonin gene-related peptide (CGRP), which are expressed mostly in unmyelinated axons, in the sensory root of the trigeminal ganglion^17,18^. We also used electron microscopic immunohistochemistry to examine the varicosities that expressed PV, SP, CGRP, VGLUT2, and the SNARE proteins syntaxin-1 and synaptosomal nerve-associated protein 25 (SNAP-25).

## Results

### Distribution of axonal varicosities within the dental pulp

**Figure 1:**
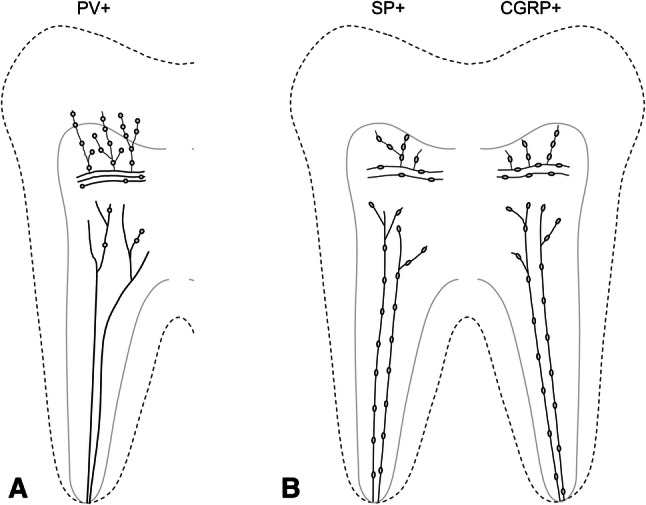
Diagrams showing the distribution of PV+ (**A**), and SP+ and CGRP+ (**B**) axons and their varicosities in the human dental pulp. (**A**) While most PV+ axons display a large number of varicosities in the peripheral pulp, few PV+ axons have any varicosities in the radicular pulp and the core of the coronal pulp. (**B**) Almost all SP+ and CGRP+ axons possess varicosities uniformly throughout the dental pulp.

**Figure 2:**
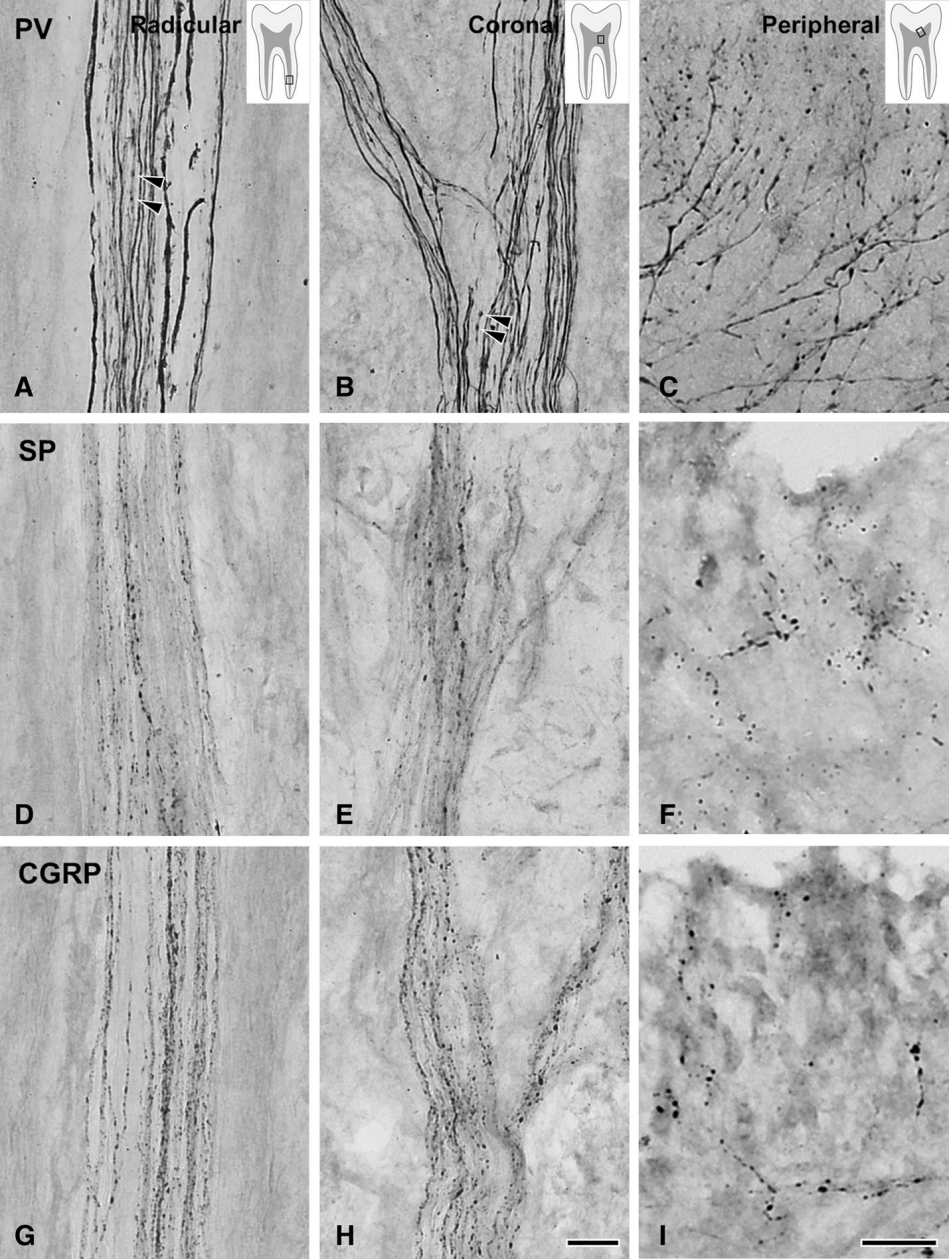
Light micrographs of PV+ (**A**–**C**), SP+ (**D**–**F**), and CGRP+ (**G**–**I**) axons in the radicular (**A**, **D**, **G**), the core of the coronal (**B**, **E**, **H**) and the peripheral (**C**, **F**, **I**) regions of the human dental pulp. Most PV+ axons have a large number of varicosities in the peripheral pulp (**C**), whereas few PV+ axons show occasional varicosities in the radicular pulp and the core of the coronal pulp (**A**, **B**). In contrast, almost all SP+ and CGRP+ axons possess varicosities throughout the dental pulp without any obvious regional differences (**D**–**I**). Arrowheads in (**A**, **B**) indicate PV+ axon varicosities in the radicular and the core of the coronal pulp. Rectangles in the insets in (**A**), (**B**), and (**C**) indicate the pulp regions represented in the respective figures. Scale bar = 20 μm (scale bar in **H** applies to **A**, **B**, **D**, **E**, **G** and in **I** applies to **C**, **F**).

Most axons in the dental pulp that were immunostained for PV, SP or CGRP were within axonal bundles in the radicular pulp and occasionally in the core of the coronal pulp. The PV-immunopositive (PV +) axons branched extensively out of these bundles and towards the dentin, whereas the SP+ and CGRP+ axons branched frequently in the peripheral pulp, distal to the parietal nerve plexus (Figs. 1, 2).

**Figure 3:**
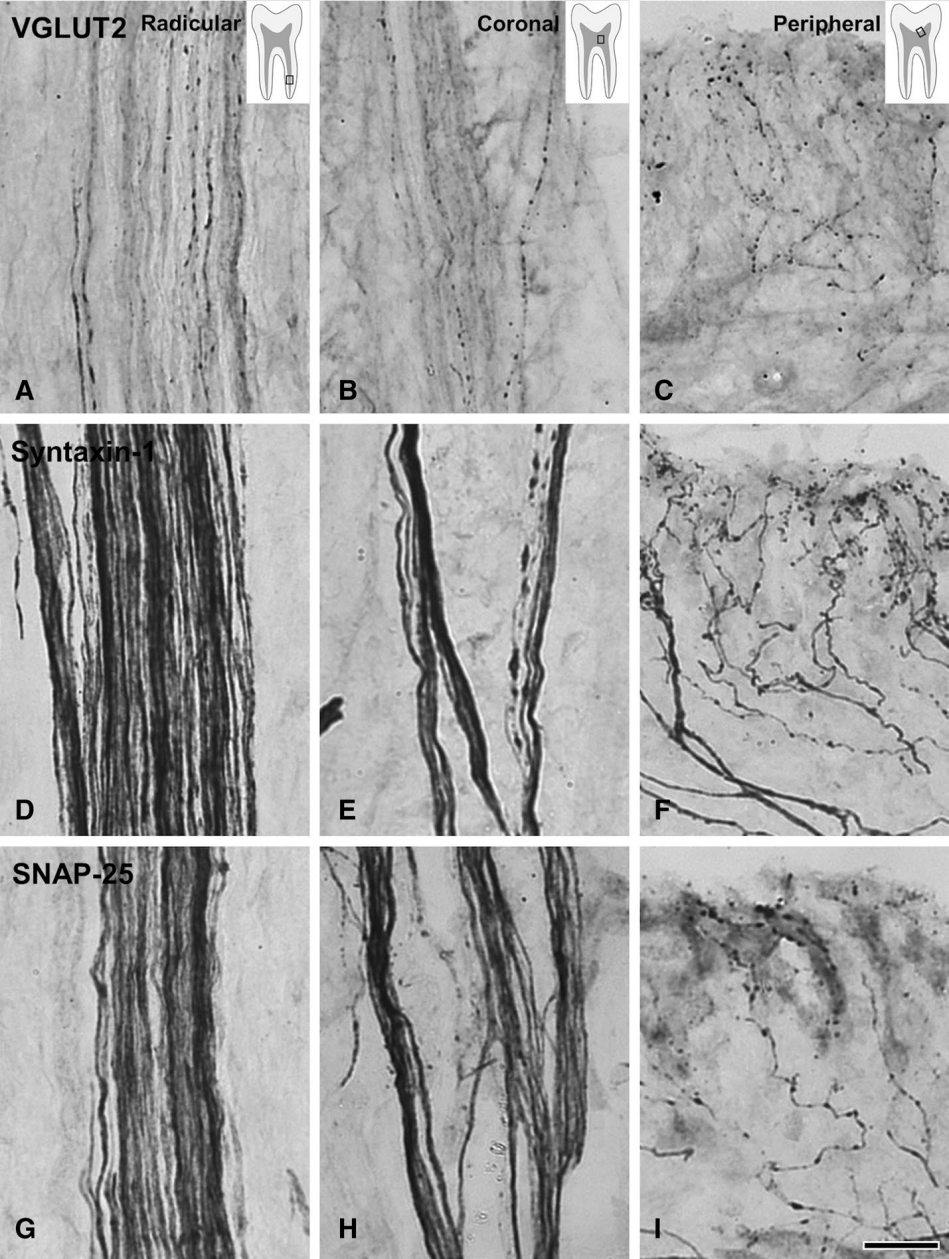
Light micrographs of VGLUT2+ (**A**–**C**), syntaxin-1+ (**D**–**F**), and SNAP-25+ (**G**–**I**) axons in the radicular (**A**, **D**, **G**), the core of the coronal (**B**, **E**, **H**), and the peripheral (**C**, **F**, **I**) regions of the human dental pulp. Most of the VGLUT2+, syntaxin-1+, and SNAP-25+ axons show numerous varicosities in the peripheral pulp. Many VGLUT2+ axons and few syntaxin-1+ and SNAP-25+ axons show varicosities in the core of the coronal and in the radicular pulp. Electron-dense immunolabeling for VGLUT2, syntaxin-1, and SNAP-25 in the axonal stands and bundles is assumed to represent the proteins that are being transported from the neuronal cell bodies to the axonal varicosities. VGLUT2+ axons show weak immunostaining (**A**–**C**), in contrast to the dense immunostaining for syntaxin-1+ and SNAP-25+ in axonal strands and bundles (**D**–**I**). Scale bar 20 μm (which applies to **A**–**H**).

**Figure 4:**
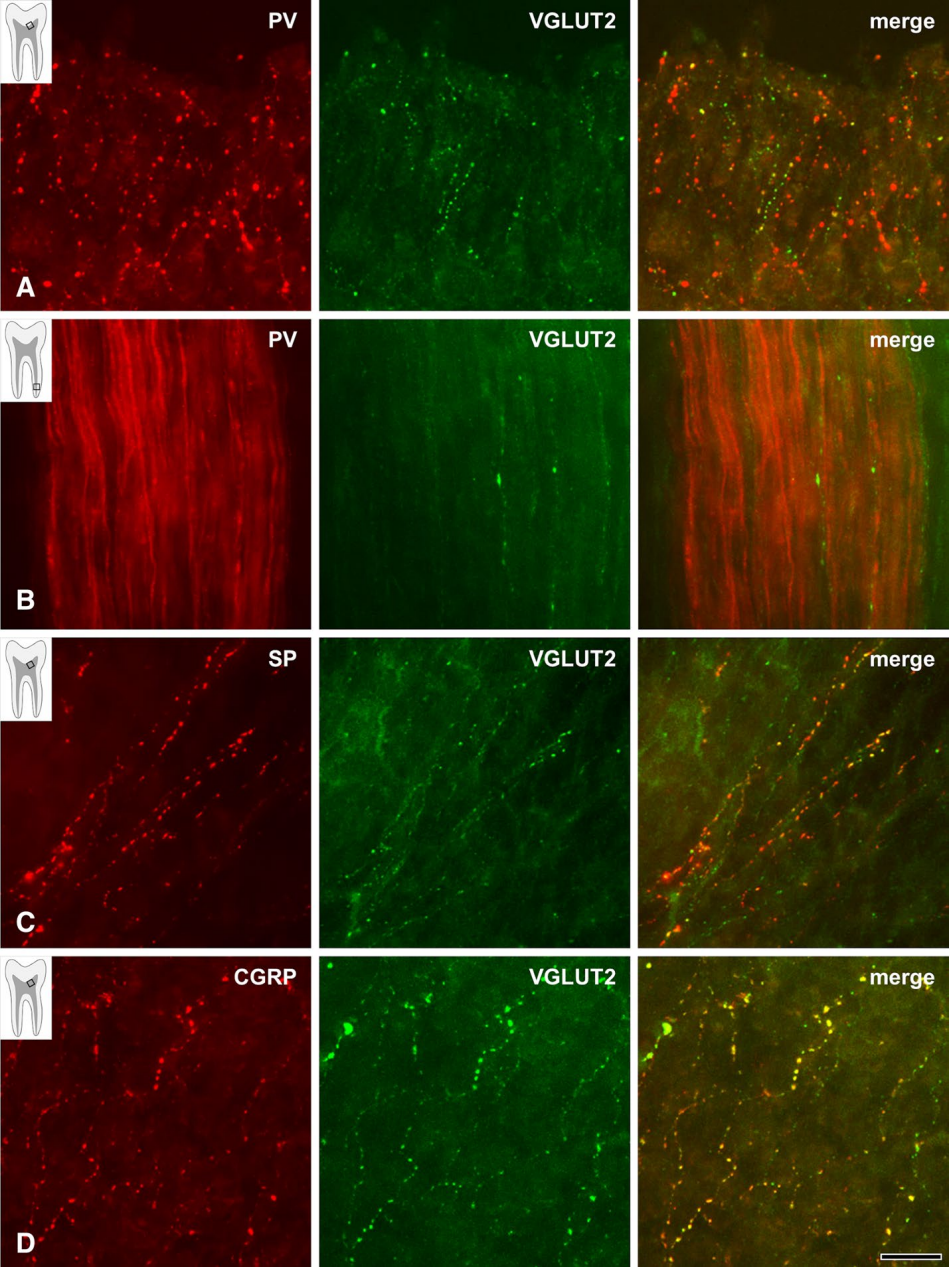
Double immunofluorescent staining for PV (red) and VGLUT2 (green) in the peripheral (**A**) and radicular (**B**) pulp, and for SP or CGRP (red) and VGLUT2 (green) in the peripheral pulp (**C**, **D**). Few PV+ axons (**A**) and the majority of SP+ (**C**) and CGRP+ (**D**) axons co-stain for VGLUT2, especially densely at their varicosities in the peripheral pulp. The PV+ axons rarely show varicosities and rarely co-stain for VGLUT2 in the radicular pulp (**B**). Insets indicate the pulp regions represented in the respective photomicrographs. Co-stain is shown in yellow in the merged images. Scale bar 20 μm.

**Figure 5:**
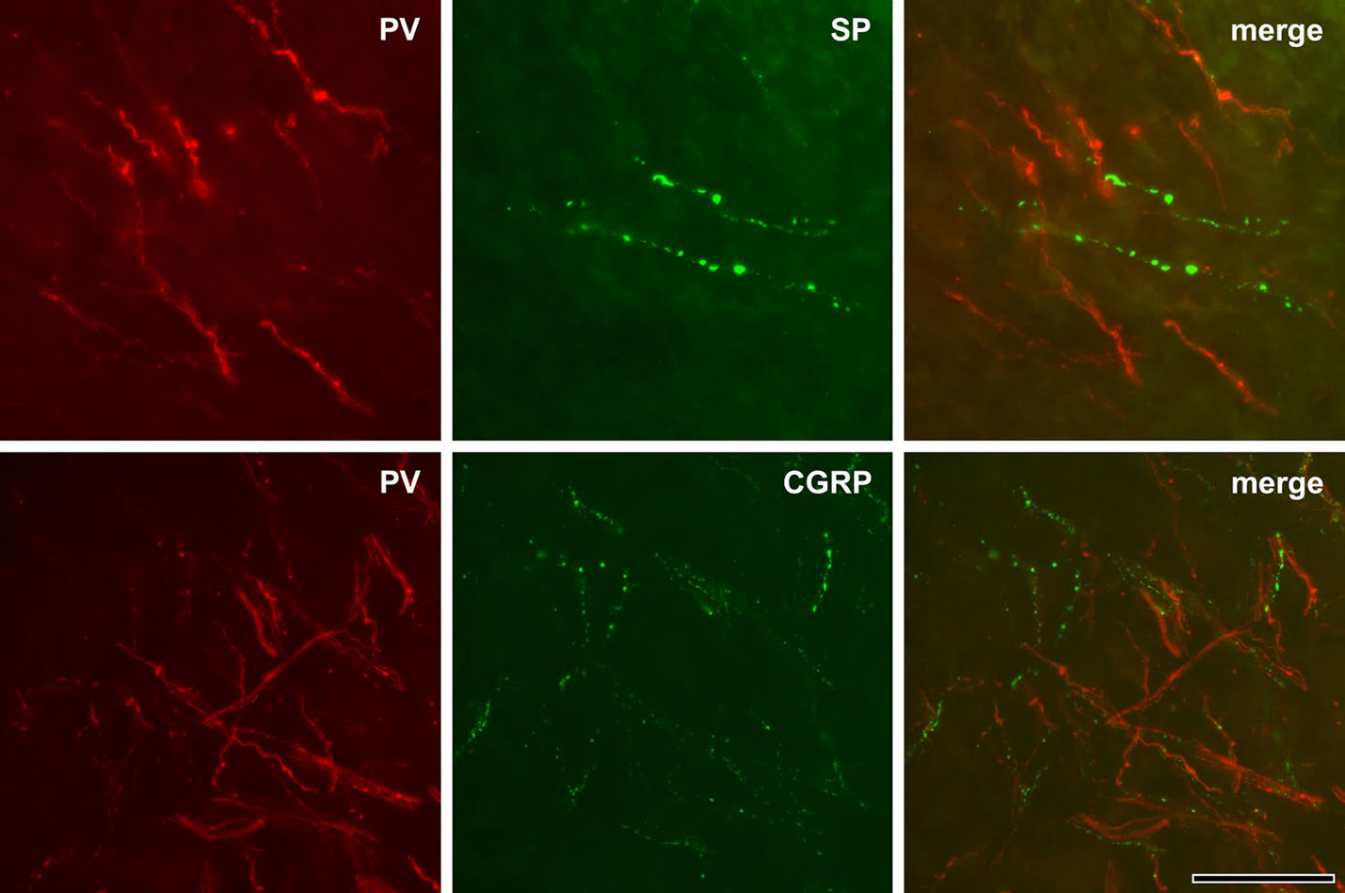
Double immunofluorescent staining for PV (red) and SP or CGRP (green) in the coronal region of the human dental pulp. PV+ axons do not co-stain for SP or CGRP. Scale bar 50 μm.

While only few PV+ axons in the radicular pulp and the core of the coronal pulp had any varicosities, virtually every PV+ axon in the peripheral pulp displayed multiple varicosities. The SP+ and CGRP+ axons, on the other hand, did not show such regional differences and had equally dense varicosities throughout the dental pulp (Figs. 1, 2). Quantitative analysis revealed that the number of varicosities per unit length of the PV+ axons was significantly higher in the peripheral pulp than in the core of the coronal and the radicular pulp. Conversely, for the SP+ and CGRP+ axons, it was similar among the three pulpal regions. The number of varicosities per unit length for PV+, SP+ and CGRP+ axons was similar in the peripheral pulp, whereas it was significantly higher for the SP+ and the CGRP+ axons than for the PV+ axons in the core of the coronal and radicular pulp (Table 1). The VGLUT2+, syntaxin-1+ and SNAP-25+ axons had numerous branches and varicosities in the peripheral pulp (Fig. 3). In the core of the coronal and the radicular pulp, many VGLUT2+ axons but few syntaxin-1+ or SNAP-25+ axons had varicosities. In addition, double immunofluorescent staining revealed that the majority of SP+ and CGRP+ axons and few of the PV+ axons expressed VGLUT2 (Fig. 4). The fraction of VGLUT2-expressing SP+ (32.6 ± 7.8%) and CGRP+ (39.2 ± 5.9%) axons was significantly higher than that of VGLUT2-expressing PV+ axons (6.2 ± 3.6%). PV+ axons did not co-stain for SP or CGRP, suggesting that in the dental pulp, PV is expressed in a different axon type than CGRP or SP (Fig. 5).

**Table 1:** Number (Mean ± SD from three pulps) of varicosities per 100 µm length of axon in 3 regions of the human pulp.

Pulpal region	PV^1^	SP	CGRP
Peripheral pulp	14.72 ± 2.61	13.66 ± 2.43	14.22 ± 0.54
Coronal pulp^2^	1.55 ± 0.25	13.02 ± 1.06	13.78 ± 0.76
Root pulp^2^	0.82 ± 0.33	11.58 ± 3.92	12.41 ± 0.96

^1^Significant difference between peripheral pulp and coronal/root pulp at the 0.01 level (one-way ANOVA, Scheffe’s test).

^2^Significant difference between PV and SP/CGRP at the 0.01 level (one-way ANOVA, Scheffe’s test).

### Ultrastructure of the axonal varicosities in the dental pulp

**Figure 6:**
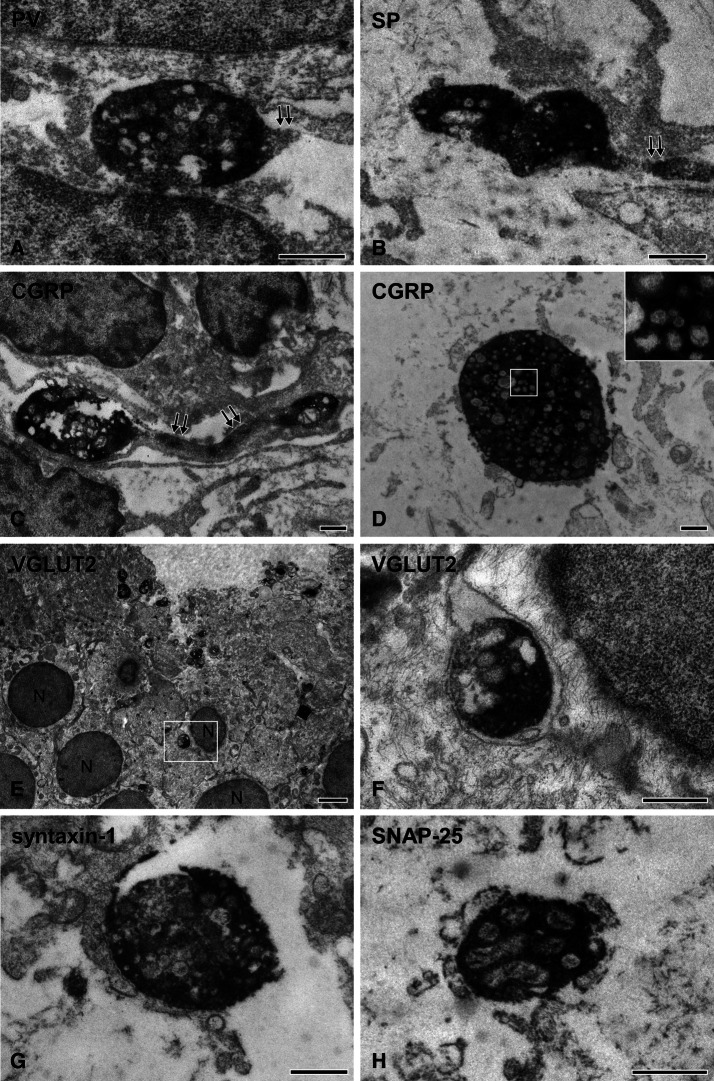
Electron micrographs showing PV+ (**A**), SP+ (**B**), CGRP+ (**C**, **D**), VGLUT2+ (**E**, **F**), syntaxin-1+ (**G**), and SNAP-25+ (**H**) axonal varicosities in the human dental pulp. The immunoreaction product is dense in varicosities but weak and patchy in the axonal segments between them (double arrows in **A**–**C**). The varicosities contain numerous small vesicles and mitochondria. Note that CGRP+ varicosities also contain large vesicles with dense cores, thought to contain neuropeptides. The insets in (**D**)is an enlargement of the boxed area within the main image. (**F**) is enlargement of the boxed area in (**E**) and shows a VGLUT2+ varicosity contacting the cell body of an odontoblast. N indicates the nucleus of the odontoblast. Scale bar 500 nm in (**A**–**D**), (**F**–**H**) and 2 μm in (**E**).

At electron microscopy, axonal varicosities were observed on unmyelinated fibers and on unmyelinated segments of myelinated fibers: Myelinated fibers entered the dental pulp as myelinated and then lost their myelin. The varicosities but not the axonal segments between them contained numerous small vesicles and mitochondria; the varicosities of SP+ and CGRP+ axons also contained large vesicles with dense cores. Varicosities were not covered by Schwann cells, which can be favorable structure for the release of neurotransmitters. The density of immunostaining for PV, SP, CGRP, VGLUT2, syntaxin-1 and SNAP-25 was significantly higher in the varicosities than in the interim segments of the axon, pointing to varicosities as a likely site of accumulation and release of glutamate and neuropeptides in the dental pulp (Fig. 6).

## Discussion

The main findings of the present study are that while the varicosities of the SP+ and CGRP+ axons were uniformly distributed throughout the human dental pulp, those of PV+ axons were only dense in the peripheral pulp, and that the expression of SP, CGRP, VGLUT2, syntaxin-1 and SNAP-25 was significantly higher in the varicosities than in the axonal segments between them. These observations support the hypothesis that axonal varicosities are sites of release of glutamate and neuropeptides by SP+ and CGRP+ unmyelinated fibers, involved in pulpal pain throughout the human dental pulp, and PV+ fibers, arising from parent myelinated fibers and involved in dentin sensitivity in the peripheral pulp only.

The axonal varicosities in the dental pulp contained vesicles and mitochondria and densely expressed VGLUT2, syntaxin-1 and SNAP-25, which are involved in vesicular loading and exocytosis of neurotransmitters and the neuropeptides SP and CGRP, which in turn, have been shown to mediate nociception and neurogenic inflammation^2–4,19^. This suggests that the axonal varicosities represent the site of release of glutamate and neuropeptides in the dental pulp, analogous to the varicosities of autonomic nerves, which also contain numerous vesicles and mitochondria, express SNARE proteins, and do not establish synaptic contact with adjacent effector cells^20,21^.

Several lines of evidence suggest that the glutamate signaling by peripheral sensory axons plays a crucial role in acute nociception and pathological pain^2–4,22^. Thus, direct application of glutamate to peripheral tissues induces excitation of nociceptive Aδ and C fibers and their subsequent sensitization^3^, and injection of glutamate into deep facial tissues evokes acute pain and induces mechanical allodynia and heat hyperalgesia^4,23,24^. In addition, it has been shown that the pulpal axons express the metabotropic glutamate receptor mGluR5 and that its expression is increased following pulpal inflammation^8,25^. By analogy, acute and pathologic dental pain may be mediated by glutamate released from the varicosities of the pulpal axons.

That the majority of SP+ and CGRP+ axons, but few of the PV+ axons coexpressed VGLUT2, which is analogous to the respective neurons in the TG^26^, suggests that the VGLUT2-dependent glutamate release in the dental pulp is mediated primarily by SP+ and CGRP+ unmyelinated axons, and to a much lesser degree by PV+ axons, arising from parent myelinated axons. Considering that the VGLUT1+ primary sensory neurons express mostly PV, NF200, and SSEA4, which are expressed in neurons with myelinated axons, and rarely CGRP and IB4, which are expressed in neurons with unmyelinated axons^26–28^, the pupal PV+ axons arising from parent myelinated axons are likely to express VGLUT1 and be involved in the VGLUT1-dependent glutamate release.

Considering that in the sensory root of TG 98.8% of the PV+ parent axons are myelinated, and 99.5% of the SP+ and 96.5% of the CGRP+ parent axons are unmyelinated^17,18^, PV+ fibers may arise from parent myelinated A fibers, and SP+ and CGRP+ fibers may arise from unmyelinated C fibers. Since it is typical for myelinated fibers to lose their myelin sheath and become thinner during their course to the peripheral target^29^, PV+ fibers lose their myelin during their course to the dental pulp. Thus, ~ 66%, 79% and 99% of the PV+ parent fibers, which are myelinated in the sensory root of the TG, become unmyelinated at the root pulp, coronal pulp and peripheral pulp, respectively^30^. Varicosities were observed within the unmyelinated segment of the PV+ fibers, but not within the their myelinated segment.

PV is expressed by a small subset of proprioceptive neurons in the dorsal root ganglion (DRG)^18,31,32^, but in a large number of low-threshold mechanoreceptive and nociceptive neurons in the TG^18,33^. Also, the human dental pulp, which has very few, if any, proprioceptors^34–36^, is densely innervated by a large number of PV+ axons^30^. Considering these findings, PV may label large number of pulpal axons arising from parent myelinated axons. SP and CGRP have been routinely used as markers for primary sensory neurons with unmyelinated fibers^37^ even though they have been shown to also label some DRG neurons with myelinated fibers^38,39^. That PV+ pulpal axons did not co-stain for SP or CGRP, which is consistent with findings in the rat dental pulp^40^, suggests that PV and SP/CGRP are expressed by distinct axon types.

In agreement with our previous observations, the PV+ axons branched extensively in the peripheral pulp distal to the parietal plexus of nerves, and many of them extended towards the dentinal tubules^30^. That almost all PV+ axons possessed numerous varicosities in the peripheral pulp but few had any varicosities in the radicular pulp and the core of the coronal pulp suggests that glutamate released from numerous varicosities of PV+ axons, following excitation in the dentin and peripheral pulp, can induce dental hypersensitivity by activating adjacent and nearby axons. This possibility is supported by the observation that numerous axonal varicosities in the peripheral pulp also express mGluR5^8^. On the other hand, that there are few PV+ axonal varicosities in the radicular pulp and the core of the coronal pulp is consistent with the observation that a considerable fraction of PV+ axons in these regions of the dental pulp are myelinated and thus do not express functional glutamate receptors and are unlikely target for either autocrine or paracrine activation and/or sensitization by glutamate^30^.

The peptidergic (SP+ or CGRP +) neurons in the TG coexpress the markers for glutamatergic transmission VGLUT^26,28,41,42^ and glutaminase^43^. We also showed previously that pulpal SP+ or CGRP+ axons frequently coexpress VGLUT2^9^. Taken together, these findings suggest that pulpal peptidergic C fibers may release glutamate as well as neuropeptides. Also, since at variance with the PV+ varicosities, the SP+ and CGRP+ varicosities were uniformly distributed throughout the dental pulp, the co-release of glutamate and neuropeptides and the subsequent autologous activation of these fibers or of adjacent fibers during inflammation, is more likely to occur uniformly throughout the dental pulp and not be confined to the peripheral pulp.

## Methods

We obtained the formal approval of the Research and Ethics Committee of the Kyungpook National University Dental Hospital prior to performing any experimental work. All experiments were carried out in accordance with the relevant guidelines and regulations. All human materials were collected for use after the subjects were explained the nature of the experiments and informed consent was obtained from all subjects or, if subjects are under 18, from a parent.

### Tissue preparation

We used maxillary premolars from nine human subjects, 15–28 years of age; the teeth were healthy and were extracted for orthodontic indication at the Department of Oral Surgery, Kyungpook National University Hospital. The pulps were extracted *in toto* by cutting the teeth along their longitudinal axes. The tissues were fixed for 5 h in a solution of 4% paraformaldehyde in phosphate buffer (PB, 0.1 M, pH 7.4) for light microscopy (6 pulps) or in a mixture of 4% paraformaldehyde and 0.01% glutaraldehyde for electron microscopy (3 pulps). The tissues were then immersed in a solution of 30% sucrose in PB at 4 °C. On the next day, 20–30 µm-thick sections for light microscopy were cut on a cryotome, and 50–60 µm-thick sections for electron microscopy were cut on a Vibratome (Leica Biosystems, Wetzlar, Germany).

### Light microscopic immunohistochemistry

Two protocols were used to prepare sections for light microscopy, immunoperoxidase for single staining and immunofluorescence for double staining. For immunoperoxidase, the sections were incubated in PB-buffered solutions of 50% ethanol for 30 min, 3% H_2_O_2_ for 10 min, and 10% normal donkey serum (NDS) (Jackson ImmunoResearch, West Grove, PA) for 10 min. They were then rinsed several times in PB and transferred to the primary antibody in phosphate-buffered saline (PBS; 0.01 M, pH, 7.4) for 18 h. We used the following primary antibodies and dilutions: mouse anti-PV (235; Swant, Marly, Switzerland) at 1:3,000, rat anti-SP (MAB356; Millipore, Billerica, MA) at 1:500, mouse anti-CGRP (ab81887; Abcam, Cambridge, MA) at 1:1,000, guinea pig anti-VGLUT2 (VGluT2-GP-Af670; Frontier Institute Co., Ltd, Hokkaido, Japan) at 1:500, mouse anti-syntaxin-1 (S0664; Sigma-Aldrich, St. Louis, MO) at 1:2,000 and rabbit anti-SNAP-25 (S9684; Sigma-Aldrich) at 1:3,000. On the next day, the sections were rinsed extensively in PBS and transferred to the appropriate secondary antibody, diluted to 1:200 in PBS for 2 h. The following biotinylated secondary antibodies were used: donkey anti-mouse, donkey anti-rat, donkey anti-guinea pig and donkey anti-rabbit (all from Jackson ImmunoResearch). Avidin–biotin-peroxidase binding was with ExtrAvidin peroxidase (Sigma-Aldrich) at 1:5,000 for 1 h. Finally, the peroxidase was revealed according to the nickel-intensified 3,3′-diaminobenzidine tetrahydrochloride (Ni-DAB) protocol. Immunostained sections were then coversliped on slides with Permount (Fisher).

For immunofluorescence, the sections were pretreated with ethanol and NDS as above and transferred to a mixture of guinea pig anti-VGLUT2 antibody (1:500) and rabbit anti-PV (1:2,000, PV 25, Swant, Marly, Switzerland), rabbit anti-SP (1:1,000, 20,064; immunostar; Hudson, WI) or mouse anti-CGRP antibody(1:1,000) or a mixture of rabbit anti-PV (1:2,000) and guinea pig anti-SP (1:1,000, AB5892, Chemicon, Temecula, CA) or mouse anti-CGRP (1:1,000) for an overnight incubation. After that, the sections were rinsed and incubated with a donkey anti-mouse or a donkey anti-rabbit antibody labeled with Cy3 in a mixture with a donkey anti-guinea pig or donkey anti-mouse antibody labeled with fluorescein isothiocyanate (Jackson ImmunoResearch) at 1:200 for 3 h. Finally, the sections were rinsed extensively and mounted on slides with Vectashield (Vector). Slides were examined on a Zeiss Axioplan 2 microscope (Carl Zeiss Inc., Jena, Germany) and a confocal microscope (LSM 510 Meta; Carl Zeiss Inc.).

### Quantitative analysis

The number of varicosities per unit axonal length was determined using sections stained with immunoperoxidase. Images were obtained at 40 × with an Ex*i* digital camera (Q-imaging Inc., Surrey, CA) attached to a Zeiss Axioplan 2 microscope (Carl Zeiss, Göttingen, Germany), and saved as TIFF files. The number of varicosities and the length of the PV+, SP+ and CGRP+ axons in each peripheral, coronal, and radicular pulp were measured from a total of 12–16 images from each pulpal region in 3–4 sections of each of three human dental pulps. Continuous strings of axonal beads with an apparent linear arrangement were considered a single axon.

The fractions of PV+, SP+ and CGRP+ axons that coexpress VGLUT2 were analyzed using sections stained with double immunofluorescence. Images were taken with a confocal microscope (LSM 510 Meta; Carl Zeiss) at 40 × at a same optical slice thickness for all channels, and saved in TIFF format. The PV+, SP+ and CGRP+ axons that coexpress VGLUT2 in the peripheral and coronal pulp were counted from a total of 12–16 images from 3–4 sections of each of three human dental pulps. All axons longer than 1 cm in the images (> 45 µm in tissue) were counted. Continuous strings of axonal beads with an apparent linear arrangement were considered to represent single axons. The differences among the three pulpal regions, and among the PV+, SP+ and CGRP+ axons was examined by one-way analysis of variance (ANOVA) and Scheffe’s F-test (significance was set at *p* < 0.05).

### Electron microscopic immunohistochemistry

To improve penetration of antibodies for electron microscopy, sections were cryoprotected in 30% sucrose in PBS overnight, frozen on dry ice for 30 min, and thawed in PBS. They were then pretreated in PBS- buffered solutions of 1% sodium borohydride for 10 min, 3% H_2_O_2_ for 10 min, and 10% NDS for 30 min. After that, the sections were transferred to a solution of the primary antibody, mouse anti-PV at 1:1,000, rat anti-SP at 1:500, mouse anti-CGRP at 1:1,000, guinea pig anti-VGLUT2 at 1:500, mouse anti-syntaxin-I at 1:2,000 or rabbit anti-SNAP-25 at 1:3,000 for an overnight incubation at room temperature. After several rinses in PBS, sections were transferred to 2% NDS for 30 min and then to an appropriate biotinylated secondary antibody, donkey anti-mouse, donkey anti-rat, donkey anti-guinea pig or donkey anti-rabbit at 1:200 for 2 h. Avidin–biotin-peroxidase binding was with ExtrAvidin peroxidase (Sigma-Aldrich) at 1:5,000 for 1 h, and peroxidase was revealed according to a standard diaminobenzidine (DAB) protocol. After additional staining in 1% osmium tetroxide in PB for 1 h, the immunostained sections were dehydrated with a series of ethanol dilutions, and embedded in Durcupan ACM (Fluka, Buchs, Switzerland). The resin was cured in a 60 °C oven for 48 h. One square mm chips of the embedded tissue were cut out and glued onto support resin blocks with cyanoacrylate. Thin sections, cut with a diamond ultramicrotome knife (Dupont), were mounted in series on formvar-coated Ni grids and the grids were counterstained with uranyl acetate and lead citrate and examined on a Hitachi H-7500 electron microscope (Hitachi, Tokyo, Japan) at 80 kV. Photomicrographs were taken with a SC1000 CCD camera (Gatan, Pleasanton, CA) and saved as TIFF files using DigitalMicrograph software.

### Immunohistochemical controls

All antibodies used in this study have been used in our laboratory and their specificity has been confirmed in previous experiments^17,18,26,30^. Nevertheless, we routinely processed sections where we omitted either the primary or the secondary antibodies. Sections incubated without the primary or secondary antibodies completely lacked specific staining. We also immunostained sections after preadsorption with blocking peptides, according to the respective supplier’s protocol: for example, sections incubated with 18 µg/mL PV blocking peptide lacked specific immunostaining for PV. With electron microscopy, we studied the immunostaining in adjacent serial thin sections: consistent staining in serial sections of the same tissue elements confirmed specificity.

## Data Availability

The datasets generated and/or analyzed in the course the current study are available from the corresponding author on request.
